# Translating Translational Research into Global Health Gains

**DOI:** 10.1371/journal.pmed.1001493

**Published:** 2013-07-30

**Authors:** The PLOS Medicine Editors

Last month, the United States Supreme Court struck down a patent claim by Myriad Genetics on the BRCA1 and BRCA2 genes [1]. The case, Association for Molecular Pathology v. Myriad Genetics, considered whether genes isolated in the lab are “products of nature” that may not be patented or are “human-made inventions” that would be eligible for patent protection. In its decision, the Court disallowed the patenting of human genes, writing that “A naturally occurring DNA segment is a product of nature and not patent eligible merely because it has been isolated” [2]. This ruling may well have considerable implications for the cost and delivery of health care, and leads us to consider just how important translational medicine is becoming to medical practice, and how far it still has to go to benefit the global community.

Translational medicine seeks to build upon basic scientific research and to translate scientific findings into medical advances that will improve clinical practice and public health. In the case above, the discovery of variants in the *BRCA1* and *BRCA2* genes that predispose individuals to breast and ovarian cancer led to the development of medical tests based on gene sequencing. Individuals with a strong family history of disease can now be tested, counseled, and potentially treated to prevent the development of disease. Because of Myriad Genetics' patent on and exclusive test for the *BRCA1* and *BRCA2* genes, individuals seeking this test were paying well above the current cost for gene sequencing; now other companies will be able to offer competing BRCA tests that may drive the costs down [3].

The potential of translational medicine to improve clinical care is reflected in two recent studies in *PLOS Medicine*. Pierre Laurent-Puig and colleagues reported on a transcriptome-based classification of colon cancer that groups colon cancers into six molecular subtypes, using clinicopathological variables and common DNA markers that are detectable due to defects in distinct biological pathways [4]. The authors of the study classified colon cancer into six different subtypes, two of which have worse outcomes overall. This classification is a significant improvement over the previous three-subtype classification, which had little prognostic value. The hope is that this new classification system can be used to help predict prognosis for stage II–III colon cancers and to identify and develop targeted treatments for the different subtypes of disease.

Another recent study published in the journal, by Nathan Ledeboer and colleagues, assessed the performance of a diagnostic platform for detecting Gram-positive bacteria in blood cultures, an important step in the diagnosis and treatment of patients with sepsis [5]. Gram-positive bacteria are the most common cause of sepsis, and rapid detection and diagnosis are critical. Currently, standard treatment of sepsis is hampered by long turnaround times to identify bacteria after culture and determine antibiotic susceptibility. In this study, the authors tested the microarray-based Verigene Gram-Positive Blood Culture (BC-GP) Test for 12 different Gram-positive bacteria and genetic markers of antibiotic resistance and demonstrate that the BC-GP Test identified Gram-positive bacteria directly from positive blood culture broths, with high sensitivity and specificity. In addition, the 2-hour turnaround time to both identify bacteria after culture and determine their antibiotic resistance could help clinicians implement rapid, effective treatment for their patients.

The promise of translational medicine has also attracted increased calls for and funding of more translational research. The UK government, for instance, announced plans to increase funding for translational medicine by 30% [6], while the US National Institutes of Health (NIH) established the National Center for Advancing Translational Sciences, with a US$575 million dollar investment, aiming to “transform the translational science process so that new treatments and cures for disease can be delivered to patients faster” [7].

With more funding, the amount of translational research is sure to increase, but to realize the potential of translational medicine, research must be of the highest possible quality. In a recent *PLOS Medicine* Essay, Carljin Hooijmans and colleagues point out several challenges in translating animal research to humans, including the poor methodological quality of some animal experiments, insufficient reporting of animal research, and publication bias. These issues may be at least as big a problem for preclinical research as they are for clinical research, where the requirements for study protocols and prospective trial registration help encourage more standardized and transparent methodology. The authors emphasize the importance of conducting systematic reviews of animal research to better support future animal and human studies and conclude that “Improving the quality and translation of animal research requires co-operation from the wider scientific community, journals, researchers, regulators, funding bodies, peer reviewers, and patients” [8]. We support this call to improve the conduct and reporting of animal research and endorse the ARRIVE guidelines for reporting of animal research [9]. Further, *PLOS Medicine* now requires authors to submit the ARRIVE checklist with all manuscripts using animal models.

Methodological issues in preclinical research are also addressed in a recent systematic review by Valerie Henderson and colleagues [10]. The authors analyzed existing guidelines for the design of preclinical research, developed an evidence-based list of methodological issues across various disciplines, and provide a researcher-friendly tool to aid in planning research. Implementing such tools can only help improve the state of the science, and ultimately perhaps a single evidence-based tool to aid researchers in the planning, conduct, and reporting of research can be developed.

The potential of translational medicine is also reduced by prohibitive expense, as exemplified in the case of the recently overturned *BRCA1* and *BRCA2* gene patents. If such testing is considered expensive in high-income countries, the tests are out of reach for most individuals in low- and middle-income countries (LMICs). Why then does *PLOS Medicine*, with our focus on global health, include a focus on translational medicine? The potential impact of translational research on global health is not a new concept. In a report from the World Health Organization on genomics and global health in 2002, commissioned after the sequencing of the human genome, the authors write, “it is clear that the science of genomics holds tremendous potential for improving health globally” [11]. If anything, LMICs have more to gain from translational medicine, where the lack of state-of-the-art laboratories make the potential for rapid, simple, low-cost diagnostics all the more compelling.

There have been some translational medicine successes for global health recently, one example of which is Xpert MTB/RIF. A PCR-based test built on the GeneXpert platform [12] and contained within a desktop machine that rapidly detects *Mycobacterium tuberculosis*, Xpert MTB/RIF can identify drug resistance in 2 hours [13]. Tuberculosis is one of the world's most serious infections, and TB cases and drug resistance are both increasing [14]. Effective diagnostic tools for TB or for detecting resistant strains had previously not been available in many highly affected areas, so Xpert MTB/RIF has significantly changed the landscape of TB diagnosis, especially for patients with HIV [15]. Xpert MTB/RIF does not require specialized laboratories or skills, and the rapid diagnosis (about 2 hours vs. weeks to test for drug-resistant TB with previous tests) is well-suited for endemic settings. However, even after the price was negotiated for high-burden countries, the cost of each machine (about USD$17,000, with extra costs for the cartridges) remains an impediment to rollout [16].

While GeneXpert and Xpert MTB/RIF are a step toward realizing the potential for translational medicine to improve global health, these advances must be affordable to realistically tackle diseases on a global scale. Governments and funding bodies have invested heavily in global public-private partnerships in the hope that vaccines, diagnostics, and treatments might be developed to benefit the global community. However, these advances must be accessible to all countries regardless of income if translational medicine is to truly improve global health and not simply widen the current inequalities in global health.

## Abbreviations

GP-BC: Gram-Positive Blood Culture
LMIC: low- and middle-income country
TB: tuberculosis
